# Supplementary material for: AluMine: alignment-free method for the discovery of polymorphic Alu element insertions
Source: Mob DNA. 2019 Jul 18;10:31. doi: 10.1186/s13100-019-0174-3 (PMC6639938; doi:10.1186/s13100-019-0174-3)
Supplement: Supplementary file 1 — Figure S1 and Figure S2 explaining the REF- and REF+ discovery algorithms. Figure S3 showing distribution of depth of coverage in tested individuals. Figure S4. A gel electrophoretic image showing the experimental validation of REF– polymorphic Alu element insertions in 48 locations and three individuals. Upper bands show the presence of an Alu insertion (alternative allele B), and lower bands show its absence (reference allele A). Predicted genotypes and expected product lengths are shown in Additional file 2: Table S5. PCR primers used for this analysis are shown in Additional file 2: Table S6. (DOCX 501 kb) [file 13100_2019_174_MOESM1_ESM.docx]

Additional File 1. Supplementary Figures

AluMine: alignment-free method for the discovery of polymorphic Alu element insertions

Tarmo Puurand, Viktoria Kukuškina, Fanny-Dhelia Pajuste and Maido Remm*

*Institute of Molecular and Cell Biology, University of Tartu, Tartu, Estonia*

**Figure S1.** **Main steps of the REF– discovery pipeline.**

Step 1. Search for 10 bp Alu signature sequences in raw reads from sequenced individuals. Extract 25 bp sequence from the 5’-flanking region of the signature sequence and add it to the 10 bp signature. Remove the candidate if the frequency of the resulting 35-mer in a given individual is <5 or >100.

Step 2. Use the 25 bp region to determine the location of the Alu element in the reference genome using gtester4. Remove the candidate if its location is not detectable in the reference genome (Alu elements from heterochromatin). Remove the candidate if the 25 bp sequence is present in multiple locations in the reference genome (Alu elements from repeated regions).

Step 3. Extract the 10 bp sequence from the reference genome. Compare it with the Alu signature. Remove the candidate if the reference genome already contains an Alu element in this position (fixed Alu elements). Element is considered as fixed if the Levenshtein distance between 10 bp from reference and 10 bp from raw reads is smaller than 3.

**Figure S2.** **Main steps of the REF+ discovery pipeline.**

Step 1. Find all 10 bp Alu signature sequences, allowing up to 1 mismatch with the reference genome.

Step 2. Identify the 5 bp target site duplication (TSD) sequence at the 5’ end of the Alu signature sequence. Search for identical 5 bp TSD sequences at the 3’ end of the Alu element. Remove the candidate if the 3’ end TSD is not detected within 270 - 350 bp of the start of the Alu signature sequence.

Step 3. Test the similarity between detected Alu elements with known Alu elements.

Step 4. Generate REF+ *k*-mers (25 nt from the genome and 7 nt from the Alu sequence) for each candidate. Count the frequencies of these *k*-mers in the chimpanzee genome, allowing 2 mismatches. Remove the candidate if the 32-mer was detected at least once in the chimpanzee genome.

**Figure S3.** **Distribution of depth of coverage values of test individuals.**

Libraries were prepared by PCR-free protocol, mean insert size 400bp. Samples were sequenced by Illumina HiSeq X Ten, read length 151 nucleotides (Mitt, 2017). Median depth of coverage was 28.1; average 28.4x; 5th percentile was 19.5x and 95th percentile was 38.5x.

**** ****

**Figure S4.** A gel electrophoretic image showing the experimental validation of REF– polymorphic Alu element insertions in 48 locations and three individuals. Upper bands show the presence of an Alu insertion (alternative allele B), and lower bands show its absence (reference allele A). Predicted genotypes and expected product lengths are shown in Supplementary Table S5. PCR primers used for this analysis are shown in Supplementary Table S6.
